# Durable and dramatic response to checkpoint inhibition combined with COX‐2 inhibitor celecoxib in a patient with p16+ metastatic sinonasal undifferentiated carcinoma: A case study

**DOI:** 10.1002/cnr2.1915

**Published:** 2023-10-22

**Authors:** Jonathan Q. Trinh, Cassaundra Acosta, Arti Easwar, Robert Galamaga, Alan Tan

**Affiliations:** ^1^ Department of Internal Medicine University of Nebraska Medical Center Omaha Nebraska USA; ^2^ Department of Medical Oncology and Hematology City of Hope Goodyear Arizona USA; ^3^ Department of Pathology and Laboratory Medicine City of Hope Goodyear Arizona USA; ^4^ Department of Internal Medicine Rush University Medical Center Chicago Illinois USA

**Keywords:** dual checkpoint inhibition, IDO1, immunotherapy, metastatic, sinonasal undifferentiated carcinoma

## Abstract

**Background:**

Sinonasal undifferentiated carcinoma (SNUC) is an exceedingly rare head and neck malignancy. No consensus exists on treatment for metastatic disease.

**Case:**

A 56‐year‐old female was diagnosed with SNUC after endorsing sinus congestion, diplopia, and right orbital pain. Initially treated with surgery and radiation, she later developed significant metastatic disease. She demonstrated progression of her hepatic metastases under pembrolizumab therapy. However, the addition of ipilimumab and a COX‐2 inhibitor resulted in significant improvement in her lesions as well as an ongoing durable response. Her regimen was complicated by immune‐related adverse events successfully treated with steroids.

**Conclusion:**

Dual checkpoint inhibition deserves consideration when treating metastatic SNUC, especially after single agent therapy has failed. The positive effect of this treatment may be augmented by IDO1 inhibition.

## 1 INTRODUCTION

Sinonasal undifferentiated carcinoma (SNUC) is a rare, poorly differentiated, and aggressive malignancy that arises from the nasal cavity and paranasal sinuses with an estimated incidence of 0.02 per 100 000 persons.^1^ Since its first description in 1986, fewer than 300 cases have been reported worldwide.^2^ Due to the rare nature of this malignancy, the literature consists of case reports and a small number of retrospective institutional series. There is no treatment consensus or guidelines for the management of SNUC, particularly in the metastatic setting.^3^

The development of immunotherapy has led to significant strides in the treatment of head and neck cancer. Pembrolizumab, a programmed cell death protein 1 (PD‐1) inhibitor, is considered first‐line therapy for unresectable recurrent or metastatic head and neck squamous cell carcinoma (HNSCC).^4^ Ipilimumab, a cytotoxic T‐lymphocyte‐associated protein 4 (CTLA‐4) inhibitor, has also recently been explored as neoadjuvant therapy in combination with nivolumab, another PD‐1 inhibitor, for HNSCC.^5^ Here we present a case of a patient with metastatic SNUC who demonstrated a durable response to treatment with both pembrolizumab and ipilimumab, which was potentially enhanced by COX‐2 inhibition via IDO1 inhibition.

## 2 CASE PRESENTATION

**FIGURE 1:**
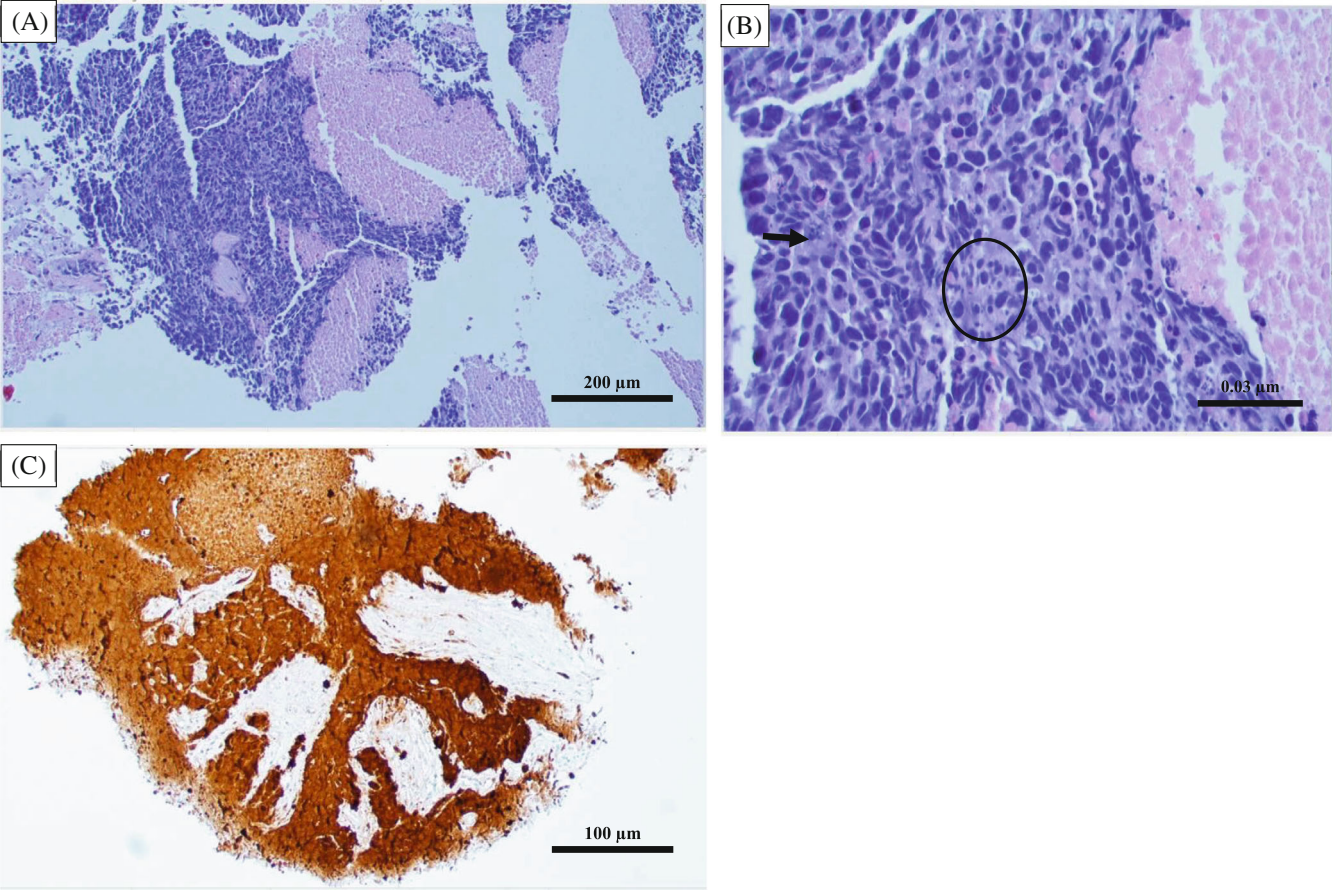
Histopathological findings in metastatic Sinonasal undifferentiated carcinoma (SNUC). Hematoxylin–Eosin (H&E) staining of liver tissue shows complete replacement of normal liver parenchyma by neoplastic high grade cells with extensive necrosis (A: ×40). The atypical cells show irregular nuclear borders, vesicular chromatin and prominent nucleoli, with abundant atypical mitoses (arrow) and apoptosis (circle) (B: ×600). Immunohistochemical stain for p16 (CINtec) is positive (nuclear and cytoplasmic staining) in the areas with viable tumor (C: ×20).

A 56‐year‐old female patient presented to the Cancer Treatment Centers of America, Goodyear, Arizona in December 2016 with complaints of sinus congestion, diplopia, and right orbital pain associated with proptosis. A magnetic resonance imaging (MRI) scan noted a 6.5 cm mass involving the right maxillary and frontal sinuses. A biopsy demonstrated a poorly differentiated neoplasm that stained positively for cytokeratin (CAM 5.2), p16, and epithelial membrane antigen (EMA), consistent with sinonasal undifferentiated carcinoma (SNUC) (Figure 1). She underwent transfacial resection including right orbital exenteration with final staging as pT4bNxM0. Due to a positive posterior margin, she underwent postoperative radiation (5940 cGy) concurrently with high‐dose cisplatin (100 mg/m^2^, 3 cycles).

**FIGURE 2:**
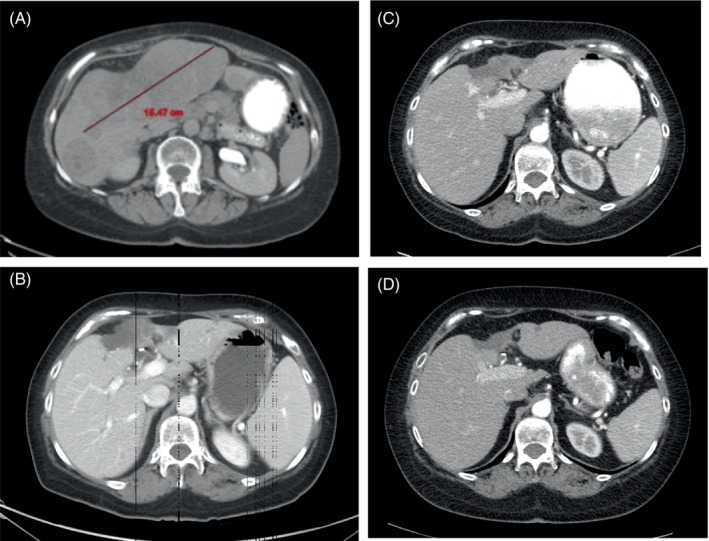
Computed tomography scans of hepatic metastases. (A) Initial presentation with largest diameter 15.5 centimeters. (B) Improvement of metastases at outside hospital after finishing treatment. (C) Continued improvement after 11 weeks from last treatment. (D) Continued improvement after 24 weeks from last treatment.

One year after initial diagnosis, she presented with cervical radiculopathy and was found to have T1 metastatic recurrence (4.5 × 2 cm) as well as extensive liver metastases (largest 15.5 × 13.8 cm) (Figure 2A). She received palliative radiation therapy to the T1 metastasis (Table 1).

**TABLE 1:** Treatment summary in chronological order.

Treatment	Date
Palliative radiation therapy to T1 metastasis	12/2016
Carboplatin and etoposide (six cycles)	12/29/2016–4/14/2017
Pembrolizumab monotherapy (four cycles)	5/3/2017–7/5/2017
Pembrolizumab and celecoxib	7/26/2017
Pembrolizumab, celecoxib, and ipilimumab	8/16/2017–9/8/2017
Pembrolizumab monotherapy	12/6/2018–2/28/2019
Stereotactic body radiation therapy	3/26/2020–4/1/2020

Next‐generation sequencing of the liver biopsy (Foundation Medicine, Cambridge, MA) revealed several genomic alterations, including equivocal amplifications of CD274 (PD‐L1) and PDCD1LG2 (PD‐L2). PD‐L1 22C3 assay resulted in a tumor proportion score (TPS) of 0%. The liver biopsy was also found to be positive for p16 and therefore potentially indicative of a human papilloma virus (HPV) infection.

The patient completed 6 cycles of carboplatin (AUC 5)/etoposide 100 mg/m^2^ days 1–3 with peg‐filgrastim support. Her best response to therapy was a 65% reduction of the largest liver target lesion. Due to mild progression after the 6th cycle of chemotherapy, single‐agent pembrolizumab 2 mg/kg every 3 weeks was started with excellent clinical tolerance; however, a computed tomography (CT) scan after four cycles demonstrated radiographic progression of liver metastases along with elevation of lactate dehydrogenase (LDH) to 1201 U/L (313–618 U/L), alkaline phosphatase (ALP) to 251 U/L (38–126), aspartate aminotransferase (AST) to 74 U/L (14–36), and alanine aminotransferase (ALT) to 80 U/L (9–52) (Table 2).

**TABLE 2:** Values of liver enzymes and LDH during treatment course.

DATE	AST	ALT	LDH	ALP
5/3/2017	38	43	483	
5/24/2017	139	192	733	
6/13/2017	57	85	808	
7/5/2017	68	62	1795	
7/25/2017	74	80	1201	251
8/16/2017	68	65	675	158
9/5/2017	73	67	507	
11/21/2017	26	34	482	101
12/6/2017	190	203		1446
12/13/2017	20	59		736
12/20/2017	11	17		406
13/30/2017	13	12		216
1/10/2018	14	12		139
1/23/2018	16	11		131
1/30/2018	16	16		132
2/14/2018	19	15		108
2/22/2018 Prednisone discontinued	36	35	495	112
2/24/2018	140	178		148

Abbreviations: ALP, alkaline phosphatase; ALT, alanine aminotransferase; AST, aspartate aminotransferase; LDH, lactate dehydrogenase.

Because of atypical clinical benefit, pembrolizumab was continued for a fifth cycle, now in combination with celecoxib 200 mg/day in hopes of suppressing constitutive expression of immunosuppressive molecule indoleamine 2,3‐dioxygenase (IDO1). During the time of progression, repeat genomic testing was done on a lymph node biopsy, which revealed the same alterations of CD274 and PDCD1LG2 though now classified as variance of unknown significance (VUS).

Three weeks after starting celecoxib, LDH decreased to 675 U/L, ALP improved to 158 U/L, and AST and ALT remained stable. Dual checkpoint inhibition with pembrolizumab 200 mg and ipilimumab 1 mg/kg was started every 3 weeks for the 6th and 7th cycles of treatment.

Three days after the last treatment she experienced grade II autoimmune dermatologic toxicity manifesting as diffuse pruritic rash, which responded to topical steroids. Six days after treatment she was admitted to an outside hospital with polyneuropathy of her upper extremities, generalized weakness, and anorexia. Additionally, she was found to have a sodium of 122 mg/dL secondary to syndrome of inappropriate antidiuretic hormone (SIADH) as well as mildly worsening liver function tests (LFTs). Celecoxib was stopped. An MRI of the brain did not show evidence of metastases but did show meningeal enhancement, concerning for leptomeningeal spread. There was also no evidence of hypophysitis, and adrenocorticotropic hormone (ACTH) and cortisol levels were normal. A lumbar puncture was negative for malignancy or infection in cerebrospinal fluid.

Due to the perceived poor prognosis and the patient's declining condition, the patient was recommended hospice care by the inpatient team. The treating oncologist, however, requested a subsequent CT scan of the liver to document true disease progression, but the imaging demonstrated marked improvement of hepatic metastases (Figure 2B). Because of this significant improvement in target lesions, it was thought that her decompensation was due to an immune‐related adverse event (irAE) rather than disease progression.

The patient was started on prednisone 1 mg/kg daily with marked improvement of her performance status and eventually improved to baseline while continuing a steroid taper over 2 months. 11 weeks after the last immunotherapy, she returned to her oncologist with a CT scan demonstrating continued improvement of liver metastases (Figure 2C). LFTs were within normal limits at this time. She did not continue immunotherapy; however, she was advised to restart celecoxib 200 mg/day. Two weeks later, she developed the onset of grade III autoimmune hepatitis manifesting as fever: ALP 1446 IU/L, AST 190, ALT 203 (Table 1). She started back on prednisone 1 mg/kg and slowly tapered to 5 mg/day.

A CT scan 24 weeks after last immunotherapy showed continued ongoing durable response with continued regression and/or stability of remaining target liver lesions (Figure 2D). LFTs were back to normal despite continuing celecoxib and low‐dose prednisone. She was then advised to stop prednisone completely and, within 2 days, had recurrence of autoimmune hepatitis (grade II): ALP 148 IU/L, AST 140 IU/L, and ALT 178 IU/L. She was advised to stop celecoxib and resume a prednisone taper, and liver enzymes remained normal thereafter.

Although the patient had oligometastatic recurrences over the subsequent years, she had good disease control and a relatively indolent course managed with single‐agent pembrolizumab plus stereotactic body radiation therapy (SBRT). Unfortunately, the patient ultimately died 4 years after she started immunotherapy due to COVID‐19 complications.

## 3 DISCUSSION

To our knowledge, this case is the first report of a response of metastatic sinonasal undifferentiated carcinoma to PD‐1/CTLA‐4 checkpoint inhibitors in combination, overcoming resistance to single agent pembrolizumab. A recent case report detailed the successful use of immunotherapy to treat a patient with metastatic SNUC.^6^ However, in contrast to the current case, that case involved the use of a single‐agent checkpoint inhibitor, nivolumab. Still, it indicated the potential use of immunotherapy for SNUC treatment, as the patient demonstrated a complete response.

Furthermore, this is also the first reported case in humans describing the potential immunomodulatory role of COX‐2 inhibition in combination with checkpoint inhibitors. COX‐2 drives the expression of IDO1, an enzyme involved in tryptophan degradation that contributes to the prevention of T‐cell infiltration in tumors and subsequent immunotherapy failure. Treatment of tumors with celecoxib, a COX‐2 inhibitor, led to reduced IDO1 levels and controlled tumor growth in mice.^7^ Our patient started celecoxib with her fifth cycle of pembrolizumab after continued progression of her liver metastases and, 3 weeks later, showed improved LDH and ALP along with stable AST and ALT. This may have been early evidence of efficacy of the combination of pembrolizumab with a COX‐2 inhibitor. Moreover, she had multiple episodes of autoimmune hepatitis while on celecoxib alone, which were responsive to systemic steroids. Therefore, celecoxib may have played an immunomodulatory role in reinvigorating this patient's immune response through IDO1 inhibition and could have contributed to her durable disease control with the combination of pembrolizumab and ipilimumab. Although the patient developed a grade IV neurologic event presenting as polyneuritis and profound weakness consistent with aseptic meningitis, she was able to safely continue single‐agent immune checkpoint inhibition with long‐term disease control. It is well‐documented that patients who develop high‐grade irAEs end up with durable disease control.^8^, ^9^, ^10^

Looking further at the genomics of the liver metastasis, it demonstrated CD274 amplification; however, there was no expression of PD‐L1 (TPS 0%). In a recent study investigating 80 tumors of oral cavity squamous cell carcinoma, 19% had *CD274/PD‐L1* gene amplification but only 73% of those cases had PD‐L1 IHC.^11^ Similarly, another study examined 118 187 tumor samples where only 0.7% had CD274/PD‐L1 gene amplification, which did not always correlate with PD‐L1 expression by IHC.^12^ They hypothesized that this could be due to post transcriptional splicing and methylation, insufficient sampling, or PD‐L2 expression rather than PD‐L1. In our case, although CD274 and PDCD1LG2 amplifications were only noted as equivocal, it is possible that they may play a role as predictive biomarkers for these tumors.

Given the nature of this report, it is important to recognize limitations. As a case study detailing one patient experience, the findings are not generalizable. Additionally, we cannot definitively conclude a causal relationship between the therapies and outcome. Ideally, experimental data would be gathered for further conclusiveness and validity. However, given the rarity of this malignancy, a formal prospective study may never be achieved.

In conclusion, this case demonstrated multiple key findings and learning points. The use of dual immunotherapy after initial failure of single agent checkpoint inhibitor to treat a rare, aggressive cancer without established treatment guidelines led to a durable response involving irAEs. Also, COX‐2 inhibition potentially contributed to this response, illustrating its promise in cancer treatment and the need to explore it further. Finally, this case underscores the importance of recognizing drug toxicity as opposed to true disease progression. In this instance, the patient was originally recommended hospice care with a perceived dismal prognosis when, in fact, her target lesions demonstrated a remarkable response. Due diligence, along with expertise in managing rare immune related adverse events; and recognition of atypical patterns of response is critical in the outcomes of such complex and rare cases.

## 4 CONCLUSION

We present, to our knowledge, the first case of metastatic sinonasal undifferentiated carcinoma (SNUC) with durable response to dual checkpoint inhibition. In addition, we showed that COX‐2 inhibition may augment this response via immunomodulatory effects on IDO1. These therapies deserve consideration as treatment options for future similar cases.

## AUTHOR CONTRIBUTIONS

**Jonathan Q. Trinh:** Writing – original draft (equal); writing – review and editing (equal). **Arti Easwar:** Visualization (equal); writing – original draft (equal). **Robert Galamaga:** Visualization (equal); writing – original draft (equal). **Alan Tan:** Investigation (equal); supervision (lead); writing – original draft (equal); writing – review and editing (equal). **Cassaundra Acosta:** Validation (equal); writing – review and editing (equal).

## CONFLICT OF INTEREST STATEMENT

The authors have stated explicitly that there are no conflicts of interest in connection with this article.

## ETHICS STATEMENT

The patient has deceased at the time of this submission, but her medical power of attorney has provided written informed consent for publication of this case, which we can provide at the request of the editors.

## Data Availability

Data sharing is not applicable to this article as no new data were created or analyzed in this study.
